# TICdb: a collection of gene-mapped translocation breakpoints in cancer

**DOI:** 10.1186/1471-2164-8-33

**Published:** 2007-01-26

**Authors:** Francisco J Novo, Iñigo Ortiz de Mendíbil, José L Vizmanos

**Affiliations:** 1 Department of Genetics, University of Navarra, 31008 Pamplona, Spain

## Abstract

**Background:**

Despite the importance of chromosomal translocations in the initiation and/or progression of cancer, a comprehensive catalog of translocation breakpoints in which these are precisely located on the reference sequence of the human genome is not available at present.

**Description:**

We have created a database that describes the genomic location of 1,225 translocation breakpoints in human tumors, corresponding to 247 different genes, using information from publicly available sources. Junction sequences from reciprocal translocations were obtained from 655 different references (either from the literature or from nucleotide databases), and were mapped onto the reference sequence of the human genome using BLAST. All translocation breakpoints were thus referred to precise nucleotide positions (949 breakpoints) or gene fragments (introns or exons, 276 breakpoints) within specific *Ensembl *transcripts.

**Conclusion:**

TICdb is a comprehensive collection of finely mapped translocation breakpoints, freely available at . It should facilitate the analysis of sequences encompassing translocation breakpoints and the identification of factors driving translocation events in human tumors.

## Background

Chromosome translocations are very important in the initiation and/or progression of cancer, and consequently a high number of translocation events have been reported in human tumors. An extensive amount of information about all types of chromosome rearrangements in cancer is stored in the *Mitelman Database of Chromosome Aberrations in Cancer *[1], a public database available at the *Cancer Genome Anatomy Project*; however, this resource does not provide molecular information beyond the name of the genes involved in each rearrangement. Although the precise genomic location of many translocation breakpoints can be inferred from published reports and nucleotide databases, a single repository where breakpoints are mapped onto the reference sequence of the human genome is not available at present. Our aim was to create such a database and provide a simple query interface, hoping that it will become a useful resource for researchers interested in analyzing sequence features in the vicinity of translocation breakpoints.

## Construction and content

We downloaded a text file containing all "Molecular Biology Associations" from the *Mitelman Database of Chromosome Aberrations in Cancer *[1], version August 2006. From this file we extracted the HUGO identifiers of all genes involved in reciprocal translocations, but not in deletions, amplifications, inversions or more complex rearrangements. This initial gene set was further refined with information from two published catalogs of genes rearranged in cancer [2,3]. We excluded immunoglobulin and T-cell receptor genes, since their breakpoint regions are already extensively covered in specific databases [4]. The final list consists of 298 genes that are involved in reciprocal translocations in hematological, mesenchymal or epithelial malignancies.

All gene models and sequences were obtained from *Ensembl *[5], version 38.36 (April 2006). *Ensembl *provides a high quality annotation pipeline [6] and stores all the information in a MySQL database from which data can be extracted automatically using the Application Programming Interface (API) provided by the *Ensembl *project [7]. Using Perl scripts, we created a text file containing the sequence of all alternative transcripts of the 298 genes, splitting the exons and introns of each transcript into separate sequences in fasta format. This file was indexed with formatdb, so that it could be used as a database for local BLAST searches [8].

We next searched *PubMed *and *Genbank *in order to find translocation junction sequences available in the public domain for all 298 genes. We identified 655 different sources of sequence data (*Genbank *records or bibliographic references) and we inspected each one of them, extracting translocation junction sequences from *Genbank *records and from the text or the figures/tables of published articles. This process yielded sequence data for 279 out of the 298 genes. All translocation junction sequences obtained are either fusion mRNAs or genomic fusion sequences.

**Figure 1:**
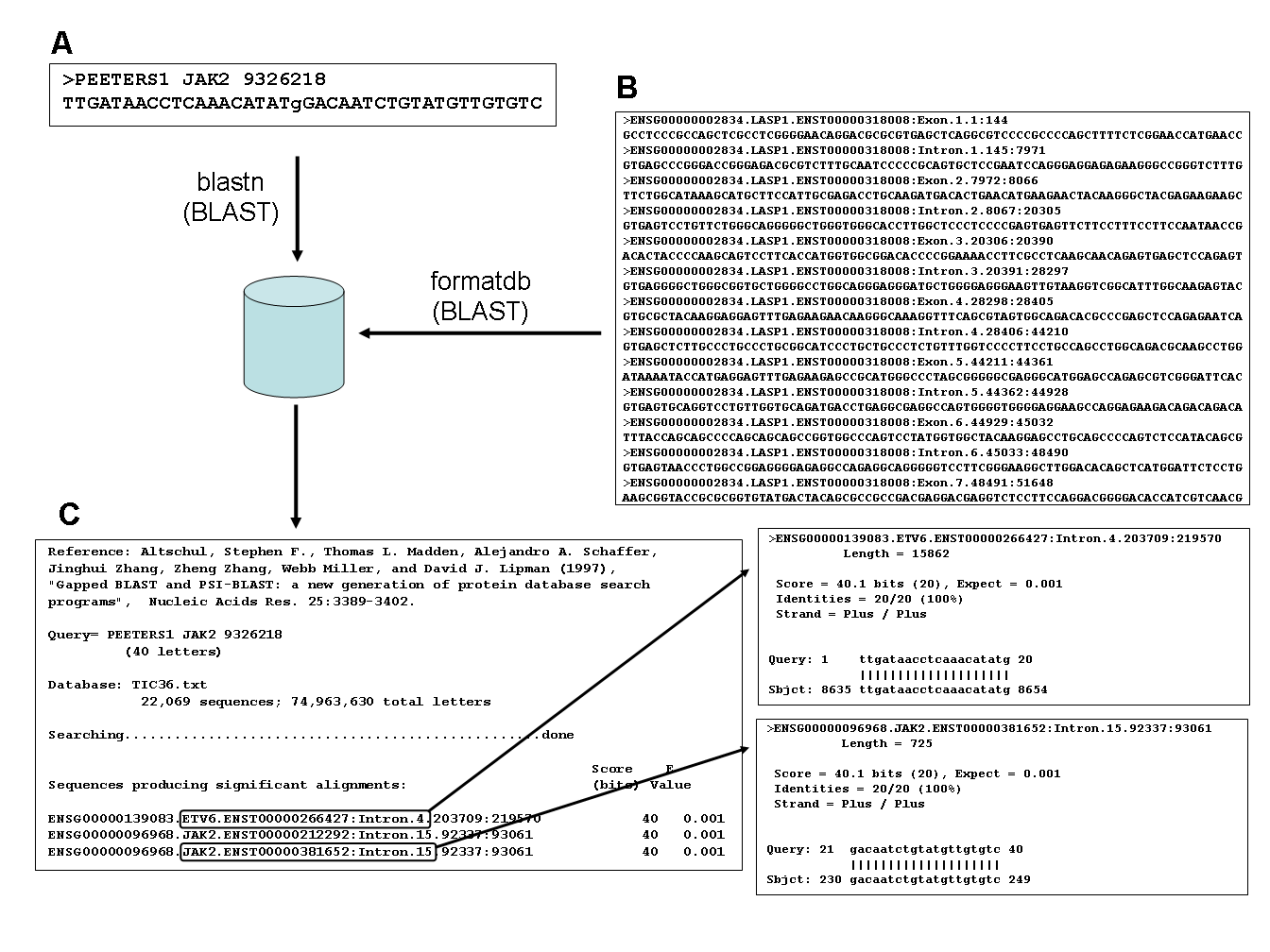
Outline of the process of mapping translocation breakpoints. Each junction sequence obtained from the literature or nucleotide database (A) was blasted against a database (B) comprising the genomic sequence of all genes known to be involved in reciprocal translocations in human tumors. Inspection of the BLAST output (C) identifies the specific region of each gene involved in the translocation. In the case of genomic fusion sequences, like the one shown in A, BLAST also identifies the nucleotide position of the breakpoint on both translocation partner genes (C, right panels). In this example the breakpoints are located in nucleotide 8654 of intron 4 of ENST00000266427 *(ETV6) *and in nucleotide 230 of intron 15 of ENST00000381652 *(JAK2)*.

The process by which translocation breakpoints were mapped onto the reference sequence of the human genome is outlined in Figure 1. Briefly, the local database containing the complete sequence of the genes was queried with each translocation junction sequence using blastn [8]. All BLAST outputs were inspected by the same database curator, in order to identify the specific intron or exon involved in each fusion. In the case of translocations for which genomic junction sequences were available, BLAST identified the exact nucleotide position of each breakpoint. When the query sequence was a fusion mRNA, BLAST matched this sequence to the exons flanking the putative intronic translocation breakpoint in each partner gene, thus establishing the identity of the specific introns involved in the translocation but not the exact position of the breakpoint. In these cases we have assumed that the breakpoint would be located in the intron preceding (or following) the exons identified by the BLAST search. The validity of this assumption is supported by the fact that 25% of these introns also contain at least one other breakpoint mapped at the nucleotide level.

Following the mapping strategy explained above we were able to map 1,225 different breakpoints, resulting from 795 different translocation events in human tumors. Furthermore, we could identify the position of 949 breakpoints at the nucleotide level of resolution. The remaining breakpoints are located to a specific fragment (an intron or an exon), but the exact nucleotide position of the breakpoint within the fragment is not known because they were mapped from mRNA fusion sequences. All these breakpoints are located in 414 unique fragments (introns or exons) belonging to 247 genes [see Additional file 2]. *Ensembl *annotations usually include several alternative transcripts for each gene. For this reason, each fragment is referred to a specific *Ensembl *transcript. The choice of transcript was based on the amount of evidence supporting each annotation: we selected transcripts manually annotated by the HAVANA group at the Wellcome Trust Sanger Institute, whenever these were available, or transcripts with an appropriate HUGO Gene Nomenclature Committee (HGNC) symbol.

**Figure 2:**
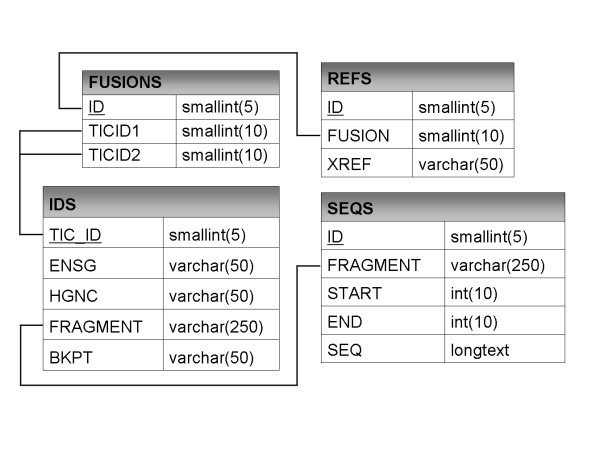
Schema of TICdb, showing the field-names and field-types for each table; underlined field names indicate primary keys.

All HGNC gene symbols, *Ensembl *gene and transcript identifiers, positions of breakpoints and fragment sequences are stored in a MySQL database. The database schema is outlined in Figure 2, which shows the structure of the tables.

## Utility and discussion

TICdb can be searched using a simple web form that allows the user to query the database by gene names. Only gene symbols approved by the HGNC are accepted; for this reason we include a list of valid HGNC gene identifiers, together with alternative aliases and a link to *Entrez Gene*, that will enable users to check gene aliases and HGNC approved names for their gene(s) of interest [see Additional file 2]. This list can also be accessed via a link to a page in which all HGNC names are hyperlinked, so that clicking on any one of them will perform the database search for that gene and take the user to the results page. This link is clearly shown in all database pages.

Searching for a valid HGNC symbol returns a table showing: i) a number identifying the fusion event; ii) for each partner gene involved in the translocation: the HGNC symbol, the description of the fragment harboring the breakpoint, and the position of the breakpoint within that fragment; and iii) the source of the junction sequence used to map the breakpoints (a cross-reference to either a *Genbank *or a *PubMed *record) hyperlinked to their respective databases. Clicking on any HGNC name in the results page will perform a database search for that gene. Fragment descriptions are in the form "ENSTX:IntronX", that is, the *Ensembl *stable identifier for the transcript, followed by the number of the intron (or exon) of that transcript containing the breakpoint. Fragment names are hyperlinked to *Ensembl Exonview*, so that the user can easily download the sequence of the fragment and locate the position of the breakpoint. Breakpoints derived from fusion transcript sequences, not mapped at the nucleotide level of resolution, are indicated as "breakpoint = 0". An Excel table with all the records present in the database sorted by gene names is provided [see Additional file 3] and is also easily accessible from the web pages. Searching with the wildcard "%" also returns all the entries in the database.

In the results page, genes are listed as 5' or 3' partner genes, depending on which part of the chimeric transcript is contributed by each gene. In this respect, it should be borne in mind that reciprocal fusion events frequently result in the generation of two chimeric transcripts, each corresponding to one of the translocated chromosomes. However, the oncogenic effect of the translocation is usually attributed to one of the fusion transcripts. For this reason, in these cases we consulted all *Pubmed *and *Genbank *sources and arranged the partner genes in the position (5' or 3') in which they appear in the fusion transcript most likely to be responsible for the disease. It should be noted that the same gene can appear as a 5' or 3' partner gene in different translocations.

The 414 unique fragments correspond to 378 introns and 36 exons, confirming that the vast majority of breakpoints (91.3%) are located within introns and that translocations very rarely disrupt exonic sequences. This is further supported by the fact that 15 of the exonic fragments that contain a translocation breakpoint are either the first or the last exon of the respective gene, with the breakpoint either located in the untranslated regions or keeping most of the coding sequence intact.

As mentioned before, all BLAST searches were manually curated. Visual inspection of BLAST outputs is necessary in order to resolve overlaps due to microhomologies, small deletions and insertions, and to choose the *Ensembl *transcript that is supported by a better annotation. This affords a high quality of the data contained in TICdb, at the cost of a rather time-consuming construction process. For this reason, general upgrades of the database will be performed only when a new NCBI build is released; regular updates including new information are much easier to do and are planned every 6 months.

The information contained in this database can be used to gain biological insights into the mechanisms leading to chromosome translocations in cancer. For instance, we have constructed a network of all the genes rearranged [see Additional file 1]. The content and topology of this network is very similar to that published by Höglund *et al*. [9], and follows a power law degree distribution. Since the network created by these authors was based on cytogenetic data, it has more nodes than our network. On the other hand, the interactions between nodes in our network are based on molecular data and so substantiate the findings of Höglund *et al*. at the molecular level.

Most importantly, TICdb should be very useful to those researchers trying to identify sequence motifs or functional and structural features associated with the appearance of a DNA double-strand break. Double-strand breaks are the initiating lesions that trigger a chromosome translocation, and the probability that a genomic region sustains a double-strand break might be dependent on its sequence context. In fact, several studies have shown that specific sequence motifs are significantly associated with translocation breakpoints in selected genes in some tumor types [10,11], but genome-wide studies have been hindered by the lack of molecular data describing the location of all published translocation breakpoints in all types of malignancies, which is precisely the information provided by TICdb. In this regard, we have previously analyzed a smaller version of this database and could identify some structural features common to all translocations in human cancer [12]. Analyses such as this were very challenging, since the data required to perform them are scattered throughout several databases or in the literature. We expect that TICdb will greatly facilitate this task and thus become a useful resource in cancer genomics.

## Conclusion

TICdb is a highly curated database containing a comprehensive catalog of translocation breakpoints in human cancers. All these breakpoints are located to specific introns or exons of *Ensembl *transcripts, many of them at the nucleotide level of resolution. Thus, TICdb constitutes an invaluable resource that should facilitate the analysis of the sequences encompassing translocation breakpoints in tumors.

## Availability and requirements

TICdb is freely available at  and has been optimized for Firefox 1.5 and Internet Explorer 6.0.

## Authors' contributions

F.J.N. and J.L.V. designed the study, F.J.N., I.O. and J.L.V. collected the data, F.J.N. analyzed the data and constructed the database. F.J.N. and J.L.V. wrote the paper. All authors read and approved the final manuscript.

## Supplementary Material

Additional File 1 A network graph showing all translocations included in TICdb, constructed using Cytoscape v2.3, showing 15 hubs (≥5 nodes/hub). The degree distribution (number of edges per node) follows a power law. Click here for file

Additional File 2 List of all 247 genes included in the database, showing the HGNC symbol, the *Ensembl *Gene identifier and other aliases. Click here for file

Additional File 3 A dump of the whole database, listing all 811 records sorted by the HGNC name of the 5' partner genes. Note that the same gene can be a 5' or a 3' partner gene, and that fusion numbers might be repeated if represented by two or more different cross-references. Click here for file
