# Supplementary figures and images for: TICdb: a collection of gene-mapped translocation breakpoints in cancer
Source: BMC Genomics. 2007 Jan 26;8:33. doi: 10.1186/1471-2164-8-33 (PMC1794234; doi:10.1186/1471-2164-8-33)

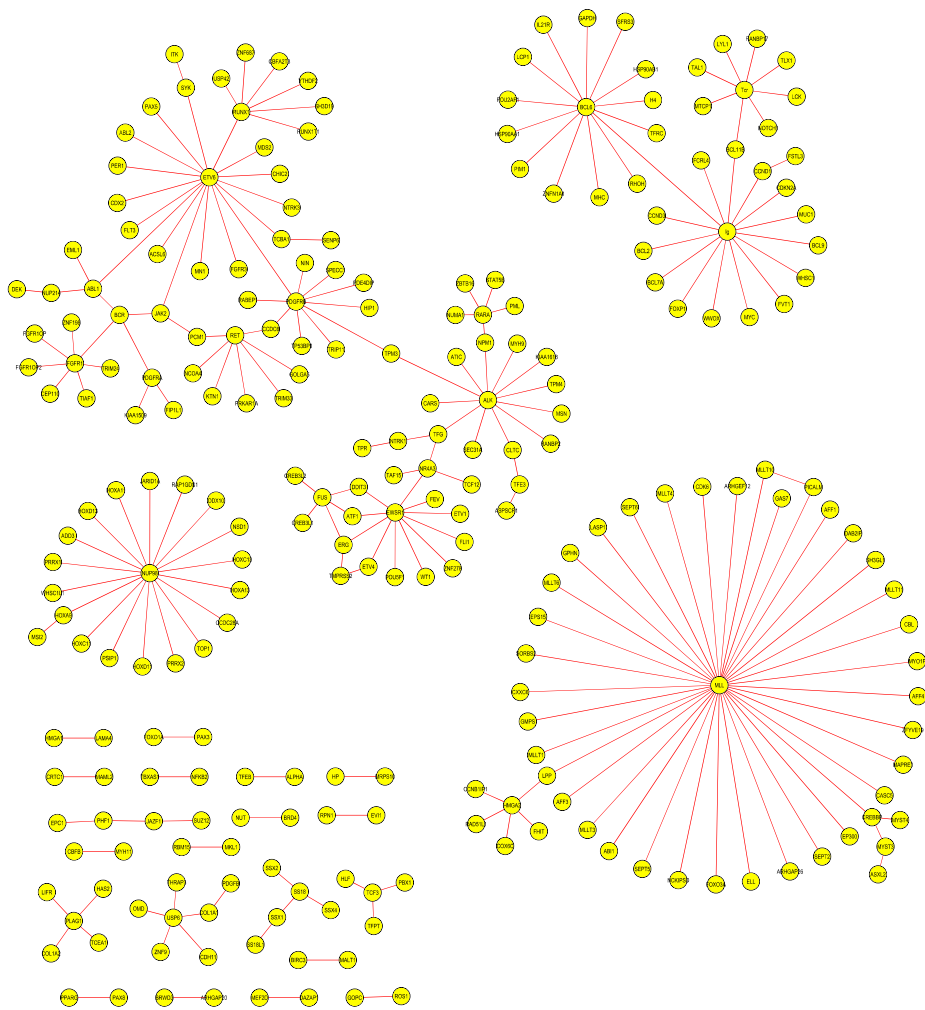

Supplement: Additional File 1 — A network graph showing all translocations included in TICdb, constructed using Cytoscape v2.3, showing 15 hubs (≥5 nodes/hub). The degree distribution (number of edges per node) follows a power law. [file 1471-2164-8-33-S1.pdf]
